# Proteomic Response to Rising Temperature in the Marine Cyanobacterium *Synechococcus* Grown in Different Nitrogen Sources

**DOI:** 10.3389/fmicb.2019.01976

**Published:** 2019-08-23

**Authors:** Yuan-Yuan Li, Xiao-Huang Chen, Cheng Xue, Hao Zhang, Geng Sun, Zhang-Xian Xie, Lin Lin, Da-Zhi Wang

**Affiliations:** ^1^State Key Laboratory of Marine Environmental Science, College of the Environment and Ecology, Xiamen University, Xiamen, China; ^2^Key Laboratory of Marine Ecology and Environmental Sciences, Chinese Academy of Sciences, Qingdao, China

**Keywords:** ocean warming, *Synechococcus*, temperature, nitrate, urea, quantitative proteomics

## Abstract

*Synechococcus* is one of the most important contributors to global primary productivity, and ocean warming is predicted to increase abundance and distribution of *Synechococcus* in the ocean. Here, we investigated molecular response of an oceanic *Synechococcus* strain WH8102 grown in two nitrogen sources (nitrate and urea) under present (25°C) and predicted future (28°C) temperature conditions using an isobaric tag (IBT)-based quantitative proteomic approach. Rising temperature decreased growth rate, contents of chlorophyll a, protein and sugar in the nitrate-grown cells, but only decreased protein content and significantly increased zeaxanthin content of the urea-grown cells. Expressions of CsoS2 protein involved in carboxysome formation and ribosomal subunits in both nitrate- and urea-grown cells were significantly decreased in rising temperature, whereas carbohydrate selective porin and sucrose-phosphate synthase (SPS) were remarkably up-regulated, and carbohydrate degradation associated proteins, i.e., glycogen phosphorylase kinase, fructokinase and glucose-6-phosphate dehydrogenase, were down-regulated in the urea-grown cells. Rising temperature also increased expressions of three redox-sensitive enzymes (peroxiredoxin, thioredoxin, and CP12) in both nitrate- and urea-grown cells. Our results indicated that rising temperature did not enhance cell growth of *Synechococcus*; on the contrary, it impaired cell functions, and this might influence cell abundance and distribution of *Synechococcus* in a future ocean.

## Introduction

The marine cyanobacterium *Synechococcus* is an abundant photosynthetic prokaryote in the ocean and contributes ∼17% of marine net primary production (Flombaum et al., 2013). Distribution and productivity of *Synechococcus* are mediated by various environmental factors. Of them, temperature is an essential factor influencing a wide range of physiology (Borbély et al., 1985), cellular processes (Borbély et al., 1985; Mackey et al., 2013), gene and protein expression (Ludwig and Bryant, 2012), and geographic distribution of *Synechococcus* (Zwirglmaier et al., 2008; Pittera et al., 2014). Recent quantitative niche models predict that ocean warming will increase cell abundance and geographic distribution of *Synechococcus*, subsequently impacting ocean ecosystem and biogeochemical cycles (Flombaum et al., 2013). Therefore, understanding the molecular mechanisms of *Synechococcus* in acclimation to ocean warming is of particular interest.

Nitrogen (N) is an essential factor for the distribution and productivity of *Synechococcus*. In estuarine and coastal waters, *Synechococcus* can use nitrate, nitrite and ammonium as the primary N source for cell growth (Rabalais et al., 2009; Wawrik et al., 2009). However, these N nutrients are extremely low throughout much of the surface oligotrophic ocean (Rees et al., 1999; Moore et al., 2013), and cannot support the growth of *Synechococcus*. Instead, urea, as the smallest organic substructure dominating the dissolved organic pool, is regarded as the main N source for cyanobacteria in the oligotrophic oceans (Solomon et al., 2010; Saito et al., 2014). Studies show that *Synechococcus* is able to utilize organic N (Moore et al., 2002; Wawrik et al., 2009; Christie-Oleza et al., 2015) and cells grown on a urea source can increase gene transcripts related to CO_2_ fixation (Ludwig and Bryant, 2012), indicating that urea has a role in controlling carbon–N flux branching between pathways.

*Synechococcus* inhabits nearly all surface oceans, from coastal waters to open ocean, crossing large scale temperature, and N nutrient gradients (Zwirglmaier et al., 2008). Despite considerable progress in understanding the response of *Synechococcus* to temperature (Ludwig and Bryant, 2012; Mackey et al., 2013; Varkey et al., 2016) or to different N sources (Moore et al., 2002; Ludwig and Bryant, 2012), little is known about the ocean warming effect on the growth of *Synechococcus* under different N conditions. It is predicted that the temperature of global ocean surface will increase 3°C on average at the end of this century due to the increasing anthropogenic activities (Albright and Mason, 2013; Schoeman et al., 2015), which will result in an increase of 14% in cell number for *Synechococcus* in the ocean (Flombaum et al., 2013). This implies that ocean warming promotes the cell growth of *Synechococcus* under different N regimes but the mechanism is not clear.

Here, we investigated growth, physiology and proteomics of an oceanic *Synechococcus* sp. strain WH8102 (hereafter WH8102), grown in two N sources (nitrate and urea) under three temperature conditions (22, 25, and 28°C) using a newly developed isobaric tag (IBT)-based quantitative proteomic approach (Ren et al., 2018). The purpose of this study was to provide insights into the acclimation mechanisms of *Synechococcus* grown in different N sources to future ocean warming. Our results demonstrated that rising temperature impaired *Synechococcus* although urea alleviated damages of rising temperature to cells to some extent, which might affect productivity and distribution of *Synechococcus* in the future ocean.

## Materials and Methods

### Cell Culture and Growth Conditions

An axenic culture of *Synechococcus* sp. WH8102 was purchased from the Bigelow Laboratory^1^ then cultured in L1 medium (Guillard and Hargraves, 1993) prepared with oligotrophic seawater obtained from the Taiwan Strait. Seawater was filtered through a micro-membrane filter and autoclave sterilization, followed by the addition of sterile nutrients (882 μM NaNO_3_ or 441 μM urea, 36.2 μM NaH_2_PO4) and EDTA-chelated metals (11.7 μM EDTA, 11.7 μM FeCl_3_, 0.9 μM MnCl_2_, 80 nM ZnSO_4_, 50 nM CoCl_2_, 10 nM CuSO_4_, 82.2 nM Na_2_MoO_4_, 10 nM H_2_SeO_3_, 10 nM NiSO_4_, 10 nM Na_3_VO_4_, and 10 nM K_2_CrO_4_) following the reported study with a few modifications (Tolonen et al., 2006). The f/2 vitamin solutions were added at final concentrations: Vitamin B1 (296 nM), biotin (2.05 nM), and Vitamin B12 (0.369 nM). 441 μM urea was added because it contains two N atoms per molecule. Cultures were grown at 22, 25, and 28°C with an irradiance of 20 μmol photons m^–2^ s^–1^ provided under a 14:10 h light: dark photoperiod.

Prior to the experiment, approximately 5 × 10^5^ cells/μL WH8102 cells were grown in fresh L1 medium at their experimental temperatures and nitrogen types, respectively. After three transfers, temperature and nitrogen-acclimated parent cells at the late exponential stage were concentrated and transferred to fresh L1 medium containing one of the following N sources: 882 μM NaNO_3_ or 441 μM urea. Each treatment was carried out in triplicate, each with a volume of 800 mL. Cell density, Fv/Fm and N nutrient concentration were monitored every day. Growth rate (*T*_d_; defined as doubling times per day) was calculated according to the Mackey et al. (2013): *T*_d_ = log_2_(*N_i_*_+ 1_/*N*_i_), while *N_i_*_+ 1_ is the cell numbers of the *i* + 1 day, *N*_i_ is the cell numbers of the *i* day. The average growth rate was calculated in the log-phase of cell growth. Late-log phase cells were harvested for physiological, RNA and quantitative proteomic analysis. It should be pointed out that data of the cells at the 22°C was absent due to the insufficient cells harvested.

### Pigment Analysis

Pigments were extracted following the protocol of Mantoura and Llewellyn (1983). Briefly, approximately 7 × 10^7^ cells was harvested and resuspended in 1 mL N, *N*-dimethylformamide (DMF) solution. After an hour incubation in −20°C and strong vortexing, the extraction was passed through Millipore syringe filters (GF/F) to remove cell debris, then mixed with the same volume of 1 M ammonium acetate. Pigment analysis was performed using a Dionex UltiMate 3000 HPLC fitted with an Eclipse XDB C8 column (4.6 × 150 mm, 3.5 μm particle size, 100 Å pore size, Agilent Technologies, Waldbronn, Germany). A UV–VIS DAD detector was used for data collection. The sample injection volume was 200 μL. Pigment identification process was followed the protocol of Zeng et al. (2016). All procedures were conducted at low temperature conditions and avoid light so as to minimize pigment decomposition.

### Protein Extraction, Digestion, and Peptide Labeling

Two biological replicates were used for protein extraction based on a reported protocol with a few modifications (Varkey et al., 2016). Approximately 7 × 10^7^ cells of each sample in the late-log phase were processed with TRIzol, chloroform, ethanol (95%) and then precipitated in ice-cold acetone at −20°C overnight. After centrifugation for 20 min at 20,000 × *g*, the protein pellet was re-suspended in rehydration buffer containing 7 M urea, 2 M thiourea, 40 mM Tris and 2% SDS, and the protein concentration was determined using the Bradford method (Bradford, 1976). For protein digestion, 100 μg protein from each sample was digested with trypsin (Promega, Madison, WI, United States) at the ratio of protein: trypsin = 40: 1 at 37°C for 12 h. Acquired peptides were treated with a demineralization process using Strata X column (Phenomenex, Torrance, CA, United States) and then dried by vacuum centrifugation, and then re-dissolved in 0.2 M riethylammonium bicarbonate (TEAB) buffer. The peptide labeling was performed using an IBT Reagent 10-plex Kit provided by the Shenzhen Genomics Institute. IBT Reagent is a new type of reagents for isobaric peptides labeling and useful in a large quantity peptides of quantitative proteomics (Xing et al., 2017; Ren et al., 2018). The labeling efficiency of peptides exceeded more than 97% at ratio of 10:1 of reagent/peptide (w/w) in 200 mM TEAB buffer for 2 h, with a wide dynamic range of 50-folds without obvious matrix effect on quantification. Briefly, 2 μg IBT reagent was thawed in 80 μL isopropyl alcohol, followed by a quick mixing with 100 μg peptides prepared in the previous step for at least 2 h at room temperature with the isopropanol concentration remained at >75%. The labeling process was stopped by adding trifluoroacetic acid (Ren et al., 2018). A total of 10 samples (five treatments with two replicates of each treatment) were used for IBT labeling and the samples were labeled as: 114 (22U_1) and 115C (22U_2) with cells grown at 22°C with urea as the sole N source; 116N (25U_1) and 117C (25U_2) with cells grown at 25°C with urea as the sole N source; 115N (25N_1) and 116C (25N_2) with cells grown at 25°C with NaNO_3_ as the sole N source; 117N (28N_1) and 118C (28N_2) with cells grown at 28°C with NaNO_3_ as the sole N source; and 118N (28U_1) and 119 (28U_2) with cells grown at 28°C with urea as the sole N source (Supplementary Figures S1, S2).

### Separation of Peptides by C18

Labeled peptides were dried and reconstituted in 2 mL buffer A (5% ACN, pH 9.8). Then, they were separated using a Gemini C18 column (4.6 mm × 250 mm, 5 μm particles, Phenomenex, Torrance, CA, United States) on a LC-20AB HPLC pump system (Shimadzu, Kyoto, Japan) at a flow rate of 1 mL/min with gradient buffer B (95% ACN, pH 9.8) at 5% for 10 min, 5–35% for 40 min, 35–95% for 1 min, and again at 5% for 10 min. The elution process was monitored at 214 nm and a total of 20 fractions were collected and dried *in vacuo*. The peptides were then dissolved in buffer C (2% ACN, 0.1% FA) and centrifuged at 20,000 × *g*. the supernate liquid was directly loaded on a trap column to remove salts and subsequently a C18 column (360 μm O.D. × 75 μm I.D.) with linear gradient buffer D (98% ACN, 0.1% FA) at 5% for 0–8 min, 8–35% for 8–43 min, 35–60% for 43–48 min, 60–80% for 48–50 min, 80% for 50–55 min, and 5% for 55–65 min at a constant flow rate of 300 nL/min.

### LC-MS/MS Analysis

The MS analysis was performed using a Q-Exactive Plus hybrid quadrupole-Orbitrap mass spectrometer (Thermo Fisher Scientific, San Jose, CA, United States) under the data-dependent acquisition mode. The electrospray voltage applied was 1.6 kV. MS scan ranged from 350 to 1500 m/z at a resolution of 30,000 and the MS/MS resolution of 30,000. Precursor ions with a 2+ to 5+ charge state and a peak strength greater than 20,000 were selected to conduct MS/MS scans. The normalized collision energy was setting at 30%. Fragment ions were detected in the Orbitrap with 30.0 s dynamic exclusion. The MS2 noise cutoff was applied at S/N of 1.5 and the signals of reporter ions at least 5% intensity of the maximum peak were accepted for quantification.

### Protein Identification and Quantification

Raw MS data were transformed through the Proteome Discoverer 1.2 (Thermo Fisher Scientific) into MGF format. Protein identification was performed using Mascot 2.3.02 (Matrix Science, Boston, MA, United States), which was then compared against the proteomic database of *Synechococcus* sp. WH8102 (2511 proteins with an additional 245 common contaminant sequences) (Palenik et al., 2003). The defaults of Mascot search were set as follows: the peptide and fragment mass tolerance were 20 ppm and ±0.05 Da. Variable modifications included Oxidation (M) and IBT 10plex (Y) while the fixed modification included Carbamidomethyl (C), IBT 10plex (N-term), and IBT 10plex (K). For IBT quantification, the Mascot results were re-estimated using the IQuant algorithm (Wen et al., 2014) with the picked protein false discovery rate (FDR) strategy (Savitski et al., 2015). To avoid bias, a missing reporter is imputed as the lowest observed values in each sample in IQuant (Kristjansdottir et al., 2013), and the imputed values could be basically evaluated by checking an output file. To reduce the signal noise caused by sample preparation and the measurement procedure, we normalized the abundances of reporter ions through variance stabilization normalization (Karp et al., 2010). At least one unique peptide with filtration of 1% FDR (PSM-level FDR ≤ 0.01; Protein level FDR ≤ 0.01) were used for follow-up quantification analysis. Non-unique peptides and outlier peptide ratios were not subjected to quantitative calculation (Tukey, 1977; Breitwieser et al., 2011). The weight approach proposed by Breitwieser et al. (2011) is employed to evaluate the ratios of protein quantity based on reporterion intensities. The ratio of the protein is calculated according to the ratio of the unique peptides, which were directly calculated by the label intensity ratio in the corresponding spectrogram. If the peptides correspond to multiple spectrograms, the average value is taken. Each protein identified had to contain at least one unique peptide and one unique spectra. Differently expressed proteins (DEPs) were defined with the criteria of *Q*-value < 0.05 and the fold change >1.5 or <0.67. For two groups of biological replicates, only proteins that were defined as differently expressed in at least one comparable group (set as 25U_1-vs-22U_1 and 25U_2-vs-22U_2, same for others) were considered to be different proteins. DEPs were clustered using hierarchical cluster software PermutMatrix (v1.9.3) (Caraux and Pinloche, 2005).

The mass spectrometry data have been deposited to the PRIDE Archive^2^ via the PRIDE partner repository with the data set identifier PXD011884.

### Physiological and Biochemical Parameter Assay

Protein concentration of 7 × 10^7^ *Synechococcus* sp. WH8102 cells was detected using a 2-D Quant Kit (GE Healthcare, Germany) at an absorption of 480 nm following the manufacturer’s instructions. Total sugars were detected according to the anthrone method (Hassid and Abraham, 1957). Briefly, frozen cells were ultrasonicated in MilliQ-H_2_O, then, a certain volume of cell lysates transferred into 80% sulfuric acid and boiled for 1 h. Anthrone reagent (0.2 g in 100 mL 80% sulfuric acid) was instantly added into the mixture, and boiled for an additional 10 min. A gradient concentration of glucose solution was carried out as described above to prepare standard curves. Samples and standards were conveyed to the detection at the absorbance of 620 nm.

Ribulose-1,5-bisphosphate carboxylase/oxygenase (RuBisCO) activity was determined following the protocol provided by the kit from Suzhou Comin Biotechnology Co., Ltd. First, the cells were ultrasonicated in lysate, and then the total protein content was measured using a 2-D Quant Kit. Next, phosphoglycerate kinase (Reagent 1) and glyceraldehyde dehydrogenase (Reagent 2) were added and the decrease rate at 340 nm absorbance within 20 s was recorded. The activity of RuBisCO carboxylase was calculated as the amount of NADH produced from 1 mg protein within 1 min.

Approximately 7 × 10^7^ *Synechococcus* WH8102 cells were stored at −80°C until the ELISA was performed. The carbonic anhydrase (CA) protein concentration was measured using a MyBiosource ELISA assay (Plant Carbonic Anhydrase ELISA Kit, Cat. No: MBS9372022). The assays were conducted according to the manufacturer’s guidelines. After incubation with HRP-Markers and thorough washing, TMB color solution was added. The absorbance of antibody-antigen-enzyme antibody complex derived from the samples and standard samples of 20, 10, 5, 2.5, 1.25, and 0.625 ng/ml were measured at 450 nm, the most commonly wavelength used for the TMB chromogenic reagent. CA content of the samples was calculated based on the standard curve and dilution multiples.

Nitrate, nitrite, and ammonium concentrations in the culture medium were measured using a Skalar San++ continuous flow analyzer^3^. The concentration of urea was determined using a colorimetric method (Chen et al., 2015). The Fv/Fm was detected using a WATER-PAM fluorometer with WinControl software (Walz) as described in Mackey et al. (2013).

### RNA Extraction and qPCR Analysis

Three biologically repeated RNA samples in the late-log phase were processed as described by Atshan et al. (2012) with modifications. Briefly, the thawed samples of approximately 7 × 10^7^ cells were lysed in TRIzol reagent and then ultrasonicated in ice bath for 10 min. The cell lysate were mixed with the chloroform of 200 μL and centrifuged at 12,000 *g* for 20 min at 4°C. The RNeasy mini Kit (Qiagen, Germany) and the QuantiTect Reverse Transcription Kit (Qiagen, Germany) were used to perform RNA purification and cDNA biosynthesis. gDNAase (provided by the Transcription Kit) was added to remove genomic DNA. The qPCR assay was performed in an ABI 7500 Thermal Cycler in a volume of 20 μL according to the SuperReal PreMix Plus Kit (Qiagen, Germany), with an initial preheating at 95°C, followed by 40 cycles of 95°C for 15 s and 60°Cfor 1 min. qPCR results were analyzed according to Li et al. (2018). The WH8102-specific primers of *urea transporter (urtA2)*, *nitrate transporter (nrtA)*, *nitrite reductase (nirA)*, *urease subunit A (ureA)*, *glutamine synthetase (glnA)*, and *glutamate synthase (glsF)* were designed using the online Primer3 Input (v0.4.0) basing on the cDNA sequences obtained from WH8102 genome (Supplementary Table S4). The expression of 16S rRNA in WH8102 was treated as internal reference. Relative quantification of gene expressions was performed using the delta-delta Ct method, as described by Livak and Schmittgen (2001). All data were expressed as the mean ± SD after normalization.

### Statistical Analysis

To estimate the statistical significance (*P*-values) of the protein quantitative ratios, IQuant adopts the permutation test (iteration 1000 times), anon-parametric approach reported by Nguyen et al. (2012). For each protein, IQuant provided a significance evaluation (*Q*-values) that is corrected for multiple hypothesis testing by the Benjamini–Hochberg method (Benjamini and Hochberg, 1995). For each physiological parameter, three biological replicates were analyzed. Statistical significance was analyzed using *t*-test performed on the IBM Predictive Analytics Software (PASW) Statistics (v18). Furthermore, the analysis of variance (ANOVA) under the general linear model was used to examine potential interactions between temperature and nitrogen for the physiological assays (Fe, 1946; Kim, 2015).

## Results

### Physiological Responses to Rising Temperature

**FIGURE 1:**
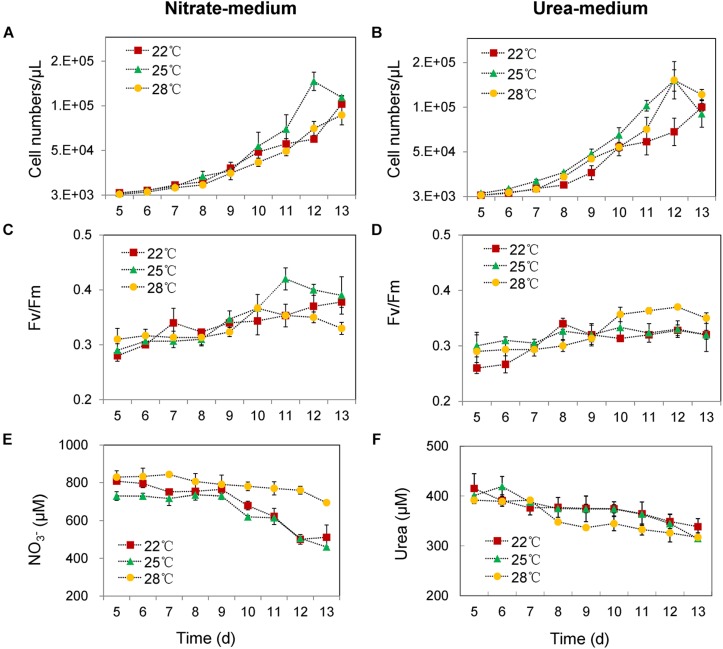
Cell numbers, photosynthetic efficiencies (Fv/Fm) and N nutrient concentrations in culture media at different growing temperatures. **(A,C,E)** With nitrate as the sole N source. **(B,D,F)** With urea as the sole N source. Red squares represent cell growth at 22°C, green triangle lines represent 25°C and yellow circle lines represent 28°C. The initial cell density was about 1000 cells/mL in all cultures, and the initial concentrations of nitrate and urea were 882 and 441 μM. Differences were very small in the first 4 days, and thus, only the changes in the following days are shown. The results are the means of three biological replicates. Error bars denote ± SD of the mean (*n* = 3).

**FIGURE 2:**
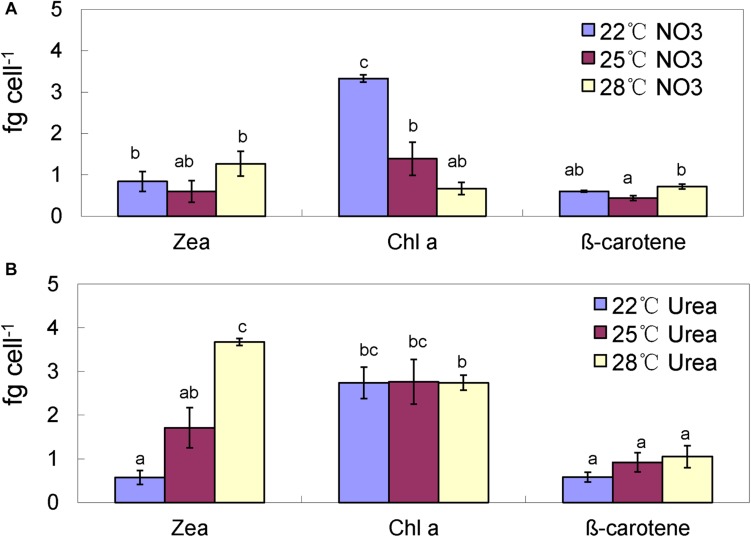
Pigment contents of the *Synechococcus* WH8102 cells at different growing temperatures. **(A)** With nitrate as the sole N source. **(B)** With urea as the sole N source. Zea, zeaxanthin; Chla, chlorophyll a. The results are the means of three biological replicates. Error bars denote ± SD of the mean (*n* = 3). Different letters indicate significant differences between treatments at *P* < 0.05.

**FIGURE 3:**
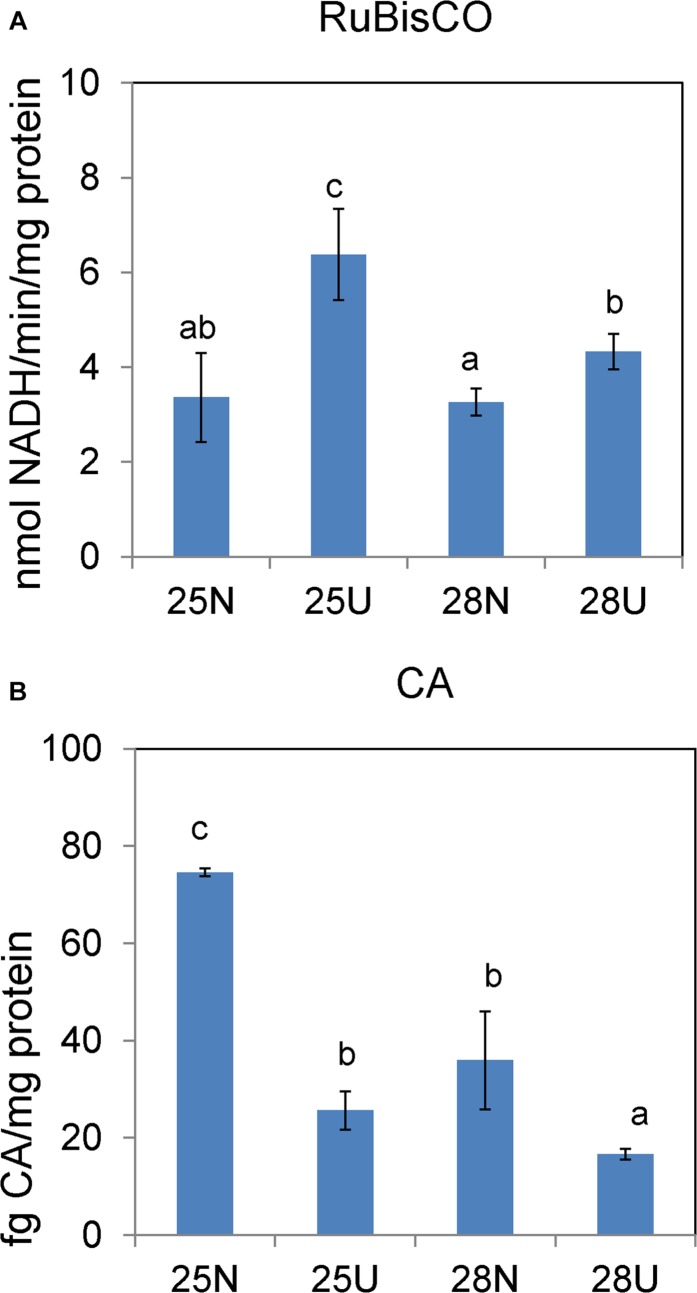
*In vitro* activities of RuBisCO carboxylase **(A)** and carbonic anhydrase **(B)** in the *Synechococcus* WH8102 cells grown on different N sources under 25 and 28°C. “N” refers to nitrate source and “U” to urea source. The results are the means of three biological replicates. Error bars denote ± SD of the mean (*n* = 3). Different letters indicate significant differences between treatments at *P* < 0.05.

In the nitrate-grown cells, cell density was higher at 25°C than that at 22°C or 28°C, and cell growth rate at 28°C was not significantly different from the rate at 22°C, but it was slightly less than that at 25°C. Whereas, they varied insignificantly in the urea-grown cells (Figures 1A,B, Table 1, and Supplementary Table S1). Photosynthetic efficiency (Fv/Fm) of the nitrate-grown cells was a little higher than that of the urea-grown cells, but it decreased at 28°C, while the urea-grown cells maintained a relatively high value of Fv/Fm at 28°C (Figures 1C,D). Nitrate uptake by cells declined at 28°C but urea uptake was enhanced (Figures 1E,F). Contents of sugar, protein and Chlorophyll a (Chl a) in the nitrate-grown cells were significantly decreased with rising temperature, while only protein content was obviously decreased in the urea-grown cells at 28°C (Figure 2 and Table 1). Content of zeaxanthin in the urea-grown cells increased significantly with rising temperature, while it varied insignificantly in the nitrate-grown cells (Figure 2). Temperature rising had no effect on β-carotene content in both nitrate- and urea-grown cells. RuBisCO activity was depressed only in the urea-grown cells (Figure 3A) while CA activity of both nitrate- and urea-grown cells was decreased with rising temperature (Figure 3B). Moreover, the urea-grown cells presented high RuBisCO activity and low CA activity compared with the nitrate-grown cells (Figure 3). Due to insufficient harvested cells, data of the cells at the 22°C was failed to be obtained. Interestingly, temperature and N sources caused significantly interactive influences on growth rate (*F* = 10.60, *df* = 2, *p* = 0.002), contents of Chla (*F* = 13.03, *df* = 2, *p* = 0.001) and zeaxanthin (*F* = 10.00, *df* = 2, *p* = 0.003), and activities of RuBsico (*F* = 11.27, *df* = 1, *p* = 0.01) and CA (*F* = 7.27, *df* = 1, *p* = 0.027). However, no significant interactive influences on total contents of proteins (*F* = 2.51, *df* = 2, *p* = 0.123) and sugars (*F* = 2.14, *df* = 2, *p* = 0.161) were observed.

**TABLE 1:** Growth rate, sugar and protein contents of the *Synechococcus* WH8102 cells grown on nitrate or urea at 22, 25, and 28°C.

**Strains**	**Temperature**	**Average growth rate (*T*_d_)**	**Total sugar content (μg/10^9^ cells)**	**Total protein content (μg/10^9^ cells)**
WH8102 + NO_3_	22°C	0.58 ± 0.03ab	10.24 ± 0.19b	1.11E^2^ ± 5.89b
	25°C	0.62 ± 0.01b	5.35 ± 0.83a	7.28E^1^ ± 4.13a
	28°C	0.53 ± 0.02a	6.51 ± 0.45a	6.32E^1^ ± 3.81a
WH8102 + urea	22°C	0.56 ± 0.03ab	19.79 ± 3.44b	1.36E^2^ ± 2.50c
	25°C	0.67 ± 0.03b	10.33 ± 1.66b	1.50E^2^ ± 5.16c
	28°C	0.63 ± 0.02b	14.16 ± 2.58b	1.10E^2^ ± 4.80b

*Each value is the mean ± SD (*n* = 3). Values not sharing the same letters are significantly different at *P* < 0.05.*

### Proteomics Overview

To explore the molecular basis of cellular responses to rising temperature, the IBT-based proteomic analysis was carried out (Supplementary Figure S2). A 2,79,923 spectra were generated and 71,837 matching maps were obtained under the 1% FDR filter. Using stringent criteria, a total of 10,856 peptides and 1764 proteins matched by at least one unique peptides were confidently identified and selected for further protein quantification (Supplementary Table S2), representing approximately 70% of the 2511 predicted proteins in the *Synechococcus* WH8102 genome (Palenik et al., 2003). Among them, 80% were identified with two or more unique peptides and 68% were with more than 10% of the sequence coverage. Total of 209 DEPs were identified from six pairwise comparisons (Supplementary Figure S3 and Supplementary Table S3).

### Proteomic Responses to Different N Sources

**FIGURE 4:**
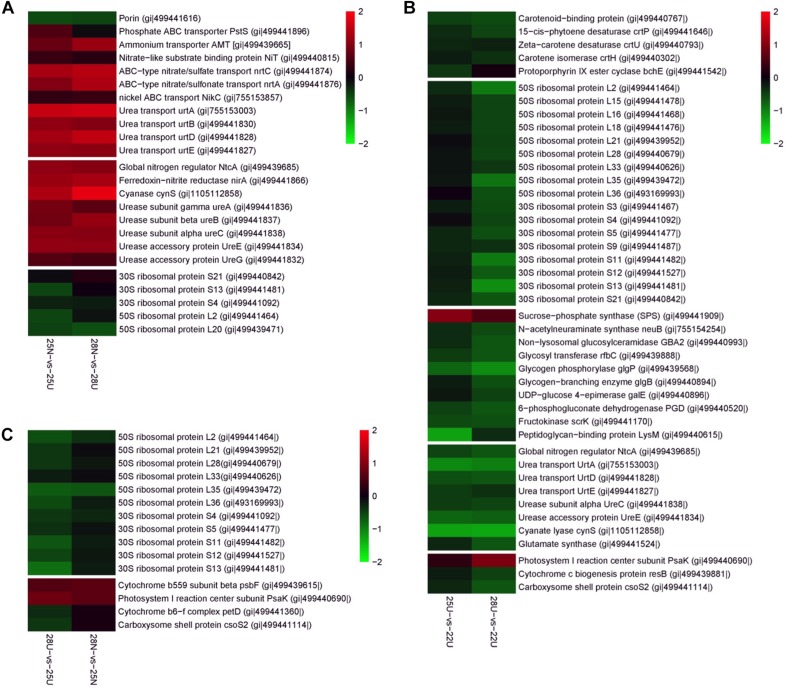
Differently expressed proteins from the *Synechococcus* WH8102 cells. Color indicates higher (red) or lower (blue) abundance relative to the centered mean value (black). Protein names and GI numbers are shown at the right. **(A)** Comparison between 25N-vs-25U and 28N-vs-28U. **(B)** Comparison between 25U-vs-22U and 28U-vs-22U. **(C)** Comparison between 28N-vs-25N and 28U-vs-25U.

The DEPs between the nitrate- and urea-grown cells were most frequently assigned to categories, including “Transporter,” “Nitrogen metabolism,” and “Urea metabolism” (Supplementary Figure S4 and Supplementary Table S3). The most relevant proteins with significant changes at each pairwise comparison were manually selected and presented in Figure 4. Global Nitrogen regulator (SYNW0275), nickel transporter (SYNW1683), four subunits of urea transporter complex (urtA, urtB, urtD, and urtE) and three subunits of urease complex (ureA, ureB, and ureC) were more abundant in the nitrate-grown cells than in the urea-grown cells (Figures 4A,C). Two accessory proteins (ureE and ureG) supporting the assembly of the nickel metallo center of urease as well as several proteins involved in inorganic N assimilation, such as ammonium transporter (AMT), nitrate transporters (nrtA, nrtC, and NiT) and ferredoxin – nitrite reductase (nirA) (Figure 4A) were also more abundant in the nitrate-grown cells.

GO term enrichment analysis further confirmed that cellular processes including “membrane” (GO: 0016020), “urease activity” (GO: 0009039), and “transporter activity” (GO: 0005478) were highly expressed in the nitrate-grown cells (Supplementary Figure S4). In addition, more cellular functions were down-regulated in the nitrate-grown cells at 25°C, including “glycosyltransferase activity” (GO: 0008194), “rRNA binding” (GO: 0019843), “Structural constituent of ribosome” (GO: 0003735), “Transferase activity” (GO: 0016758), “RNA binding” (GO: 0003723), and “Nucleic acid binding” (GO: 0003676). However, they varied insignificantly in both nitrate- and urea-grown cells at 28°C (Supplementary Figure S5).

### Proteomic Responses to Rising Temperature

Rising temperature caused changes in the abundance of proteins involved in “Nitrogen metabolism,” “Starch and sucrose metabolism,” and “Carotenoid biosynthesis” in the urea-grown cells (Supplementary Figure S4 and Supplementary Table S3). Abundances of three urea transporter subunits (urtA, urtD, and urtE) and two urease subunits (ureC and ureE) in the urea-grown cells decreased 1.53- to 2.48-fold at 25 and 28°C compared with cells at 22°C (Figure 4B). Abundance of sucrose-phosphate synthase (SPS) responsible for converting starch to soluble sugars, increased 1.68- and 2.18-fold in the urea-grown cells at 25 and 28°C compared with cells at 22°C, whereas, the abundances of three proteins involved in carbohydrate degradation, glycogen phosphorylase (glgP), 6-phosphogluconate dehydrogenase (PGD), and fructokinase (scrK), decreased 1.53- to 2.24-fold (Figure 4B). Another most frequently detected protein involved in carbohydrate transport, “Porin” protein, together with two closely linked regulators, RpaA, and RpaB was significantly up-regulated in the urea-grown cells at 28°C. Proteins involved in “Carotenoid biosynthesis,” including phytoene desaturase (crtP), isorenieratene synthase (crtU), and carotene isomerase (crtH), were down-regulated in the urea-grown cells as the temperature rose (Figure 4B).

Two enzymatic antioxidants, peroxiredoxin (Prx) and thioredoxin (Trx), and a redox-sensitive protein CP12, along with a few DNA damage-inducible proteins and molecular chaperones were significantly up-regulated in both nitrate- and urea-grown cells with rising temperature (Table 2). Five ribosomal subunits were down-regulated in the nitrate-grown cells at 25°C while 17 ribosomal subunits were down-regulated in the urea-grown cells at 28°C (Figure 4B). Notably, a carboxysome shell protein, CsoS2 involved in carboxysome formation, was down-regulated with rising temperature in both nitrate- and urea-grown cells (Figure 4C).

**TABLE 2:** Up-regulated proteins related to oxidative stress responses in the cells grown in nitrate or urea at 22, 25, and 28°C.

**Accession number**	**Name**
gi|499439633|	Thioredoxin
gi|499440416|	Peroxiredoxin
gi|499440854|	CP12 family protein
gi|499439769|	Metallothionein
gi|499441613|	Molecular chaperone DnaJ
gi|499441754|	HSP20 family protein
gi|499440429|	Ribonuclease III family protein
gi|499440145|	DNA damage-inducible protein
gi|499441456|	DNA recombination/repair protein RecA

### Validation of DEPs Using qPCR

**FIGURE 5:**
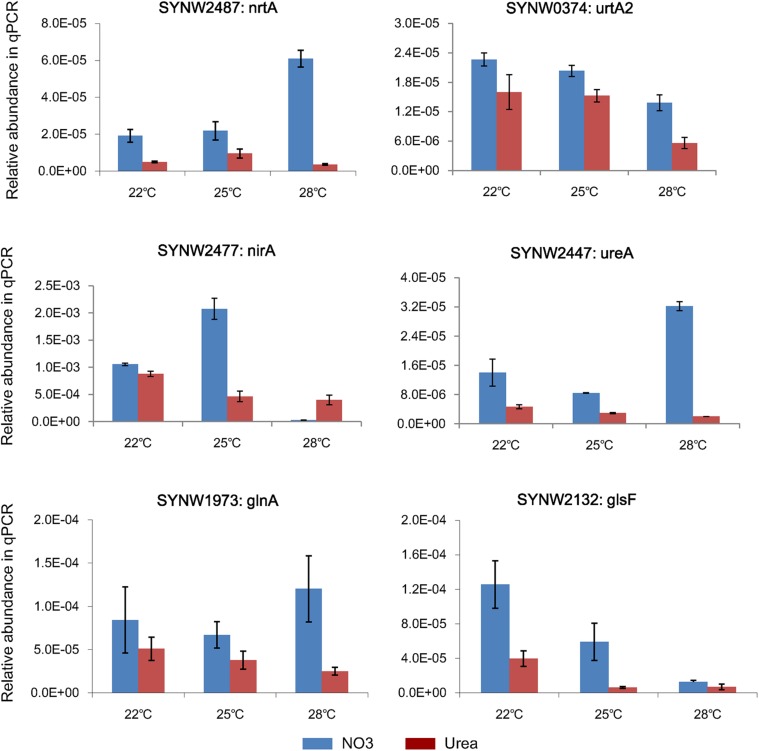
Relative transcripts of selected genes from N metabolism in *Synechococcus* WH8102.

Representative genes involved in N metabolism were selected for qPCR analysis to validate the corresponding protein expressions (Supplementary Table S4). As showed in Figure 5, expressions of *nrtA* (SYNW2487) and *ureA* (SYNW2447) in the nitrate-grown cells were remarkably increased at 28°C, while expressions of *urtA2* (SYNW0374), *nirA* (SYNW2477), and *Glutamate synthase* (*glsF*, SYNW2132) were down-regulated in both nitrate- and urea-grown cells when temperature increased to 28°C. Expression of *Glutamine Synthetase* (*glnA*, SYNW1973), which is required for ammonium assimilation, showed insignificant differences between the nitrate- and urea-grown cells under different temperature conditions. Notably, the expressions of almost all N metabolic genes in the nitrate-grown cells were higher than that in the urea-grown cells. Expression of *nirA* was decreased in the nitrate-grown cells at 28°C, which was not consistent with the variation of protein abundances. Different temporal expressions of gene and protein might be the main reason (Greenbaum et al., 2003).

## Discussion

**FIGURE 6:**
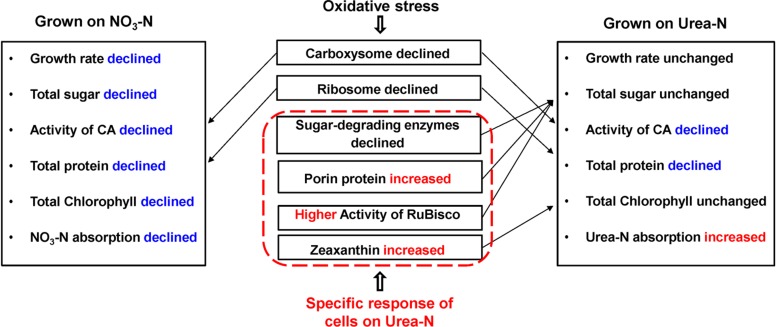
Physiological and proteomic responses of the *Synechococcus* WH8102 cells between different types of N sources under temperature increasing conditions. Growth rate of cells at 28°C on nitrate was declined when compared to cells at 25°C, but was not significantly declined at 22°C. The activity of RuBisco was declined in cells grown on urea at 28°C, but was still higher than that of cells grown on nitrate. Rates of N absorption was not assayed in the study, instead, we used the volumes of N reduction in the medium to represent the volumes of N absorbed into the cells.

Our physiological results indicated that rising temperature did not increase cell growth and biomass of *Synechococcus* grown in either nitrate- or urea condition. Accordingly, interactions between temperature and N sources significantly influenced the physiological performances. Cells grown on nitrate were more sensitive to rising temperature since lower cellular content of Chl a was observed (Figures 1A,C, 2, 6), in accord with a previous study that the elevated temperature of above 27°C leads to photo damage and results in significantly decreasing of both growth rate and photosynthetic yield (Mackey et al., 2013). However, they varied insignificantly or remained relatively high values in the urea-grown cells at 28°C (Figure 1D). In this study, urea promoted cells to synthesize more sugars and proteins when compared with nitrate, which has been considered as the inherent differences between N sources (no interactive influences were found between temperature and N sources), but it was not affected by the temperature rise, suggesting that *Synechococcus* grown in urea had stronger temperature-resistant capacity than that grown in nitrate.

Interestingly, the content of zeaxanthin in the urea-grown cells was significantly increased as temperature rose but there was no change in the nitrate-grown cells (Figures 2, 6). Carotenoids are prevalently distributed in *Synechococcus* while beta-carotene and zeaxanthin as two principal carotenoids (Six et al., 2004). Zeaxanthin not only functions as an accessory light-harvesting pigment transferring energy to chlorophyll, but also plays an important role in preventing photo-damage through quenching and dissipating the energy as heat (Zhu et al., 2010; Hashimoto et al., 2016). Previous studies show that urea promotes production of carotenoid in eukaryotic microalgae (Casal et al., 2011) and *Flavobacterium* (Bhosale and Bernstein, 2004), and the content of zeaxanthin is enhanced by ultraviolet-B radiation (Götz et al., 1999) and light stress (Six et al., 2004; Schäfer et al., 2006). Increasing zeaxanthin content in the urea-grown cells might assist cells to efficiently cope with negative impacts caused by high temperature.

Expressions of both genes and proteins involved in urea utilization were up-regulated in the nitrate-grown cells, exhibiting an NtcA-dependent manner (Figures 4A, 5). NtcA plays a governing role in up-regulating genes for N transport and assimilation of *Synechococcus* (Su et al., 2006). Inducible up-regulation of urea utilization in the nitrate-grown cells indicated that the WH8102 cells had evolved the preferential use of reducing N sources regulated by the transcriptional factor NtcA owing to living long-term in the oligotrophic environment where nitrate concentration is negligible (Palenik et al., 2003; Christie-Oleza et al., 2015). A co-expression pattern of NtcA and N utilizing proteins was observed and the expressions of *urtA2* and *ureA* were down-regulated in the urea-grown cells, although they were clearly required for urea utilization (Figures 4B, 5). Generally, nutrient limitation results in the increase of mRNA or the protein levels of nutrient uptake genes. Since the cellular uptake of N-atoms was increased in the urea-grown cells while it was decreased in the nitrate-grown cells (Figures 1E,F), down-regulation of NtcA, N-metabolic proteins and genes at 28°C in the urea-grown cells indicated that rising temperature exerted negative impacts on the nitrate-grown cells for N assimilation but promoted the absorption of N in the urea-grown cells (Figure 5).

Carbohydrates, as the main source of energy in cells, derive mainly from oxygenic photosynthesis (Matthews et al., 1999). Though cells grew at the highest rate at 25°C, few PBS pigment proteins and photosynthetic complexes were up-regulated compared with that at 22°C, which differed from the previously reported temperature-dependent protein expressions but consisted with the observation that both growth rate and Fv/Fm value are reduced when temperature exceeds 24°C (Mackey et al., 2013). Cyanobacteria generally have the effective photosynthetic CO_2_ concentrating mechanism (CCM) that enables photosynthesis to proceed smoothly (Badger et al., 2002; Iancu et al., 2007). Carboxysome, the center of the CCM, contains RuBisCO and CA which inter converts CO_2_ and HCO_3_^–^ species (Ting et al., 2015). The urea-grown cells presented high activity of RuBisCO and low activity of CA compared with the nitrate-grown cells (Figure 3), suggesting that cells grown on urea could have a less demand for CO_2_ and higher carboxylation efficiency. However, the abundances of RuBisCO and CA varied insignificantly, except that the abundance of CAs increased 1.4 fold in the urea-grown cells compared with the nitrate-grown cells at 25°C. The carboxysome shell protein, CsoS2, was significantly down-regulated in both nitrate- and urea-grown cells with rising temperature (Figures 4B,C, 6). CsoS2 protein is essential for carboxysome assembly and interacts with the shell proteins and RuBisCO (Roberts et al., 2012; Cai et al., 2015). These results indicated that ocean warming impaired carboxysome and CAs which decreased the ability of *Synechococcus* to take up ambient CO_2_, but CO_2_ produced by the hydrolysis of urea might create a suitable microenvironment for RuBisCO with high CO_2_ concentration, and thus lessened the cell requirement for outer CO_2_/bicarbonate (Mobley et al., 1995; Ludwig and Bryant, 2012), which compensated for the negative effects caused by carboxysome shell degradation.

Proteomic data also unveiled an alternative way beneficial for carbohydrate accumulation in the urea-grown cells: the urea-grown cells increased in abundance of the proteins responsible for sugar accumulation, such as SPS and porins, whereas, decreased in abundance of those proteins involved in carbohydrate degradation, such as glgP, PGD, and scrK (Figures 4B, 6). The porins, belonging to the OprB family, are mainly involved in carbohydrate uptake, including glucose, mannitol, glycerol, and fructose as reported in *Synechococcus* (Warr et al., 1985) and *Pseudomonas aeruginosa* (Wylie and Worobec, 1995). OprB can be synthesized under the osmotic stress caused by high temperature through the OmpR family (Warr et al., 1985; Wylie and Worobec, 1995; Adewoye and Worobec, 1999; Cai et al., 2015). Therefore, up-regulation of porins enhanced carbohydrate transport and accumulation, which in turn benefited the urea-grown cells so as to relieve osmotic stress caused by high temperature.

Nitrogen is essential for protein synthesis and urea allows cells to redirect saved equivalents to produce more proteins as reported (Qian et al., 2016). However, our results indicated that rising temperature caused negative effects on cellular proteins and ribosomal subunits in both nitrate-and urea-grown cells (Figures 4A,B and Table 1). Inspiringly, within the temperature range of this study, we found that ribosomes in the urea-grown cells were less readily degradable (Figure 4): total protein and ribosomal subunits were initially decreased in abundance in the nitrate-grown cells at 25°C, whereas it occurred at 28°C in the urea-grown cells. Ribosomes are the main location of protein biosynthesis and new ribosomes are continuously generated to meet the needs of the cells (Woodson, 2008). The tertiary assembly of ribosome is temperature-dependent (Powers and Noller, 1995; Dutca et al., 2007) and degradation occurs at a temperature markedly higher than the optimal growth temperature (Mangiantini et al., 1965; Saunders and Campbell, 1966; Borbély et al., 1985). Our results suggested that the negative impacts brought by ocean warming on *Synechococcus* growth were unavoidable, and ribosomal down-regulation could be the primary cause for the decrease of intracellular protein content. We postulated that the ribosome could serve as a potential indicator of a high temperature response, which was similar to the result in *Escherichia coli* upon heat shock stimuli (Van Bogelen and Neidhardt, 1990).

A previous study shows that autotrophic organisms are able to perform photosynthetic efficiency over a range of temperature surrounding the optimal growth temperature (*T*_opt_) (Mackey et al., 2013). Higher-*T*_opt_ can cause an imbalance between photochemistry and metabolism, resulting in reactive oxygen production and photo-damage as induced by light stress (Huner et al., 1996; Zhu et al., 2010; Mackey et al., 2013). For example, *Synechococcus* PCC 6301 synthesizes only a limited number of polypeptides upon a heat-shock (Borbély et al., 1985), while transcripts of genes associated with major metabolic pathways are reduced and different chaperons are dramatically increased in *Synechococcus* PCC 7002 (Ludwig and Bryant, 2012). In our study, Prx, Trx, and CP12 were significantly up-regulated in both nitrate- and urea-grown cells. Prx and Trx belong to the antioxidant defense system and the dithiol-disulfide redox regulatory network in plant and cyanobacteria (Dietz, 2011). In *Synechococcus* PCC 7942, thioredoxin-peroxidase is essential for the detoxification of H_2_O_2_ under oxidative stress (Perelman et al., 2003). CP12 is found in most photosynthetic organisms, including cyanobacteria, diatoms and high plants (López-Calcagno et al., 2014). The thioredoxin-mediated glyceraldehyde-3-phosphate dehydrogenase-CP12-phosphoribulokinase complex can protect the Calvin cycle related enzymes from oxidative damage (Marri et al., 2013). The *Synechococcus* WH8102 cells experienced oxidative stress under the rising temperature condition, however, whether it led the decline of growth rate and chlorophyll content in the nitrate-grown cells stills needs further study.

Interestingly, the zeaxanthin content of the urea-grown cells was increased significantly, suggesting that zeaxanthin might function as an antioxidant against oxidative stress derived from rising temperature. Generally, zeaxanthin biosynthesis is catalyzed by six enzymes as outlined previously (Götz et al., 1999). Promotion of urea in the production of zeaxanthin is reported (Bhosale and Bernstein, 2004; Casal et al., 2011). However, in our study, three proteins (CrtP, CrtU, and CrtH) involved in carotenoid biosynthesis (Sajilata et al., 2008; Zhu et al., 2010) in the urea-grown cells were down-regulated with rising temperature (Figure 4B), presenting a contradiction between protein expression and zeaxanthin content. Alternative pathways of zeaxanthin biosynthesis (McDermott et al., 1974), posttranscriptional and metabolite feedback regulations of carotenoid accumulation (Cuttriss et al., 2007; Bai et al., 2009; Cazzonelli and Pogson, 2010) might be responsible for this inconsistency. Most likely, as the N source, urea might provide more pigment precursors for carotenoid biosynthesis. Alcantara and Sanchez (1999) reported that asparagine and glutamine, as well as some carbohydrate catabolites, can enhance zeaxanthin production. In the urea-grown cells, the excess ammonium was transformed into asparagine and glutamine, and subsequently participated in the production of intermediates in the citric acid cycle. It is postulated that this assimilation pathway of ammonia not only promotes the synthesis of antioxidant zeaxanthin, but also reduces the toxicity of ammonia (Sakamoto et al., 1998). This hypothesis still needs further study.

## Conclusion

In summary, our results indicated that rising temperature did not enhance cell growth of *Synechococcus* grown in either nitrate or urea; on the contrary, it impaired cells regarding carbon fixation, protein synthesis and N assimilation, although urea relieved the high temperature stress to cells to a certain extent through increasing protective pigment content and more effective carbon fixation. Considering the significantly interactive influences of rising temperature and N sources on the physiological performance, we should be more cautious when we use climate change models to predict the future tendency of *Synechococcus* in the ocean regarding ocean warming, and N sources should be integrated into the prediction models. It should be pointed out that we conducted this study using the oceanic *Synechococcus* strain under high N nutrient conditions, which might not fully reflect the actual situations of natural marine environments. A comprehensive assessment of ocean warming effects on distinct *Synechococcus* strains under different N sources and levels should be carried out in both laboratory and natural environments to understand the productivity and distribution of *Synechococcus* in the future ocean.

## Data Availability

All datasets generated/analyzed for this study are included in the manuscript and the Supplementary Files.

## Author Contributions

D-ZW and Y-YL conceived the study, designed the experiment, and wrote the manuscript. Y-YL, X-HC, GS, and CX conducted the experiment and generated the data. D-ZW, Y-YL, HZ, and Z-XX analyzed and interpreted the data. LL contributed to the analytic instruments.

## Conflict of Interest Statement

The authors declare that the research was conducted in the absence of any commercial or financial relationships that could be construed as a potential conflict of interest.

## Footnotes

1 https://ncma.bigelow.org/

2 http://www.ebi.ac.uk/pride/archive/

3 https://www.skalar.com/

## References

[B1] Adewoye L. O. Worobec E. A. (1999). Multiple environmental factors regulate the expression of the carbohydrate-selective OprB porin of *Pseudomonas aeruginosa*. *Can. J. Microbiol.* 45 1033–1042. 10.1139/w99-110 10696483

[B2] Albright R. Mason B. (2013). Projected near-future levels of temperature and pCO2 reduce coral fertilization success. *PLoS One* 8:e56468. 10.1371/journal.pone.0056468 23457572 PMC3572969

[B3] Alcantara S. Sanchez S. (1999). Influence of carbon and nitrogen sources on *Flavobacterium* growth and zeaxanthin biosynthesis. *J. Ind. Microbiol. Biotechol.* 23 697–700. 10.1038/sj.jim.2900688 10455504

[B4] Atshan S. S. Shamsudin M. N. Lung L. T. Ling K. H. Sekawi Z. Pei C. P. (2012). Improved method for the isolation of RNA from bacteria refractory to disruption, including S. aureus producing biofilm. *GENE* 494 219–224. 10.1016/j.gene.2011.12.010 22222139

[B5] Badger M. R. Hanson D. Price G. D. (2002). Evolution and diversity of CO2 concentrating mechanisms in cyanobacteria. *Funct. Plant Biol.* 29 161–173. 10.1071/PP01213 32689463

[B6] Bai L. Kim E. H. DellaPenna D. Brutnell T. P. (2009). Novel lycopene epsilon cyclase activities in maize revealed through perturbation of carotenoid biosynthesis. *Plant J.* 59 588–599. 10.1111/j.1365-313X.2009.03899.x 19392686

[B7] Benjamini Y. Hochberg Y. (1995). Controlling the false discovery rate: a practical and powerful approach to multiple testing. *J. R. Stat. Soc. B Methodol.* 57 289–300. 10.1111/j.2517-6161.1995.tb02031.x

[B8] Bhosale P. Bernstein P. S. J. (2004). β-Carotene production by Flavobacterium multivorum in the presence of inorganic salts and urea. *J. Ind. Microbiol. Biotechnol.* 1 565–571. 10.1007/s10295-004-0187-9 15592945

[B9] Borbély G. Surányi G. Korcz A. Pálfi Z. (1985). Effect of heat shock on protein synthesis in the cyanobacterium *Synechococcus* sp. strain PCC 6301. *J. Bacteriol.* 161 1125–1130. 3918983 10.1128/jb.161.3.1125-1130.1985 PMC215016

[B10] Bradford M. M. A. (1976). Rapid and sensitive method for the quantitation of microgram quantities of protein utilizing the principle of protein-dye binding. *Anal. Biochem.* 72 248–254. 10.1006/abio.1976.9999 942051

[B11] Breitwieser F. P. Muller A. Dayon L. Kocher T. Hainard A. Pichler P. (2011). General statistical modeling of data from protein relative expression isobaric tags. *J. Proteome Res.* 10 2758–2766. 10.1021/pr1012784 21526793

[B12] Cai F. Dou Z. Bernstein S. L. Leverenz R. Williams E. B. Heinhorst S. (2015). Advances in understanding carboxysome assembly in *Prochlorococcus* and *Synechococcus* implicate csoS2 as a critical component. *Life* 5 1141–1171. 10.3390/life5021141 25826651 PMC4499774

[B13] Caraux G. Pinloche S. (2005). PermutMatrix: a graphical environment to arrange gene expression profiles in optimal linear order. *Bioinformatics* 21 1280–1281. 10.1093/bioinformatics/bti141 15546938

[B14] Casal C. Cuaresma M. Vega J. M. Vilchez C. (2011). Enhanced productivity of a lutein-enriched novel acidophile microalga grown on urea. *Mar. Drugs* 9 29–42. 10.3390/md9010029 21339944 PMC3039468

[B15] Cazzonelli C. I. Pogson B. J. (2010). Source to sink: regulation of carotenoid biosynthesis in plants. *Trends Plant Sci.* 15 266–274. 10.1016/j.tplants.2010.02.003 20303820

[B16] Chen L. Ma J. Huang Y. Dai M. H. Li X. L. (2015). Optimization of a colorimetric method to determine trace urea in seawater. *Limnol. Oceanogr. Methods* 13 303–311. 10.1002/lom3.10026

[B17] Christie-Oleza J. A. Armengaud J. Guerin P. Scanlan D. J. (2015). Functional distinctness in the exoproteomes of marine *Synechococcus*. *Environ. Microbiol.* 17 3781–3794. 10.1111/1462-2920.12822 25727668 PMC4949707

[B18] Cuttriss A. J. Chubb A. C. Alawady A. Grimm B. Pogson B. J. (2007). Regulation of lutein biosynthesis and prolamellar body formation in *Arabidopsis*. *Funct. Plant Biol.* 34 663–672. 10.1105/tpc.010302 32689394

[B19] Dietz K. J. (2011). Peroxiredoxins in plants and cyanobacteria. *Antioxid. Redox Signal.* 15 1129–1159. 10.1089/ars.2010.3657 21194355 PMC3135184

[B20] Dutca L. M. Jagannathan I. Grondek J. F. Culver G. M. (2007). Temperature-dependent RNP conformational rearrangements: analysis of binary complexes of primary binding proteins with 16S rRNA. *J. Mol. Biol.* 368 853–869. 10.1016/j.jmb.2007.02.064 17376481 PMC2265208

[B21] Fe S. (1946). An approximate distribution of estimates of variance components. *Biometrics* 2 110–114. 20287815

[B22] Flombaum P. Gallegos J. L. Gordillo R. A. Rincón J. Zabala L. L. Jiao N. (2013). Present and future global distributions of the marine Cyanobacteria Prochlorococcus and *Synechococcus*. *Proc. Natl. Acad. Sci. U.S.A.* 110 9824–9829. 10.1073/pnas.1307701110 23703908 PMC3683724

[B23] Götz T. Windhövel U. Böger P. Sandmann G. (1999). Protection of photosynthesis against ultraviolet-B radiation by carotenoids in transformants of the cyanobacterium *Synechococcus* PCC7942. *Plant Physiol.* 120 599–604. 10.1104/pp.120.2.599 10364412 PMC59299

[B24] Greenbaum D. Colangelo C. Williams K. Mark G. (2003). Comparing protein abundance and mRNA expression levels on a genomic scale. *Genome Biology.* 4:117. 10.1186/gb-2003-4-9-117 12952525 PMC193646

[B25] Guillard R. R. L. Hargraves P. E. (1993). Stichochrysis immobilis is a diatom, not a chrysophyte. *Phycologia* 32 234–236. 10.2216/i0031-8884-32-3-234.1

[B26] Hashimoto H. Uragami C. Cogdell R. J. (2016). Carotenoids and photosynthesis. *Sub Cell. Biochem.* 79 111–139. 10.1007/978-3-319-39126-7-4 27485220

[B27] Hassid W. Z. Abraham S. (1957). Chemical procedures for analysis of polysaccharides. *Methods Enzymol.* 3 34–50. 10.1016/s0076-6879(57)03345-5

[B28] Huner N. P. A. Maxwell D. P. Gray G. R. Savitch L. V. Krol M. Ivanov A. G. (1996). Sensing environmental temperature change through imbalances between energy supply and energy consumption: redox state of photosystem II. *Physiol. Plant* 98 358–364. 10.1034/j.1399-3054.1996.980218.x

[B29] Iancu C. V. Ding H. J. Morris D. M. Dias D. P. Gonzales A. D. Martino A. (2007). The structure of isolated *Synechococcus* strain WH8102 carboxysomes as revealed by electron cryotomography. *J. Mol. Biol.* 372 764–773. 10.1016/j.jmb.2007.06.059 17669419 PMC2453779

[B30] Karp N. A. Huber W. Sadowski P. G. Charles P. D. Hester S. V. Lilley K. S. (2010). Addressing accuracy and precision issues in iTRAQ quantitation. *Mol. Cell. Proteomic.* 9 1885–1897. 10.1074/mcp.M900628-MCP200 20382981 PMC2938101

[B31] Kim T. K. (2015). T test as a parametric statistic. *Korean J. Anesthesiol.* 68 540–546. 10.4097/kjae.2015.68.6.540 26634076 PMC4667138

[B32] Kristjansdottir B. Levan K. Partheen K. Carlsohn E. Sundfeldt K. (2013). Potential tumor biomarkers identified in ovarian cyst fluid by quantitative proteomic analysis, iTRAQ. *Clin. Proteomic.* 10:4. 10.1186/1559-0275-10-4 23557354 PMC3637236

[B33] Li Y. Y. Chen X. H. Xie Z. X. Li D. X. Wu P. F. Kong L. F. (2018). Bacterial diversity and nitrogen utilization strategies in the upper layer of the northwestern pacific ocean. *Front. Microbiol.* 9:797. 10.3389/fmicb.2018.00797 29922238 PMC5996900

[B34] Livak K. J. Schmittgen T. D. (2001). Analysis of relative gene expression data using real-time quantitative PCR and the 2(-Delta Delta C(T)) Method. *Methods* 25 402–408. 10.1006/meth.2001.1262 11846609

[B35] López-Calcagno P. E. Howard T. P. Raines C. A. (2014). The CP12 protein family: a thioredoxin-mediated metabolic switch? *Front. Plant Sci.* 5:9. 10.3389/fpls.2014.00009 24523724 PMC3906501

[B36] Ludwig M. Bryant D. A. (2012). *Synechococcus* sp. Strain PCC 7002 transcriptome: acclimation to temperature, salinity, oxidative stress, and mixotrophic growth conditions. *Front. Microbiol.* 3:354. 10.3389/fmicb.2012.00354 23087677 PMC3468840

[B37] Mackey K. R. Paytan A. Caldeira K. Grossman A. R. Moran D. McIlvin M. (2013). Effect of temperature on photosynthesis and growth in marine *Synechococcus* spp. *Plant Physiol.* 163 815–829. 10.1104/pp.113.221937 23950220 PMC3793060

[B38] Mangiantini M. T. Tecce G. Trentalance T. A. (1965). A study of ribosomes and of ribonucleic acid from a thermophilic organism. *Biochim. Biophys. Acta.* 2 252–274. 10.1186/s12864-015-1239-4 5319744

[B39] Mantoura R. E. C. Llewellyn C. A. (1983). The rapid determination of algal chlorophyll and carotenoid pigments and their breakdown products in natural waters by reverse phase high-performance liquid chromatography. *Anal. Chim. Acta.* 151 297–314. 10.1016/s0003-2670(00)80092-6

[B40] Marri L. Thieulin-Pardo G. Lebrun R. Puppo R. Zaffagnini M. Trost P. (2013). CP12-mediated protection of calvine benson cycle enzymes from oxidative stress. *Biochimie* 97 228–237. 10.1016/j.biochi.2013.10.018 24211189

[B41] Matthews C. E. Holde K. E. V. Ahern K. G. (1999). *Biochemistry*, 3rd Edn San Francisco: Benjamin Cummings.

[B42] McDermott J. C. B. Brown D. J. Britton G. Goodwin T. W. (1974). Alternative pathways of zeaxanthin biosynthesis in a *Flavobacterium* species. *Biochem. J.* 144 231–243. 10.1042/bj1440231 4462583 PMC1168490

[B43] Mobley H. L. Island M. D. Hausinger R. P. (1995). Molecular biology of microbial ureases. *Microbiol. Rev.* 59 451–480. 7565414 10.1128/mr.59.3.451-480.1995 PMC239369

[B44] Moore C. M. Mills M. M. Arrigo K. R. Berman-Frank I. Bopp L. Boyd P. W. (2013). Processes and patterns of oceanic nutrient limitation. *Nat. Geosci.* 6 701–710. 10.1038/ngeo1765

[B45] Moore L. R. Post A. F. Rocap G. Chisholm S. W. (2002). Utilization of different nitrogen sources by the marine cyanobacteria *Prochlorococcus* and *Synechococcus*. *Limnol. Oceanogr.* 47 989–996. 10.4319/lo.2002.47.4.0989

[B46] Nguyen H. Wood I. Hill M. (2012). A robust permutation test for quantitative SILAC proteomics experiments. *J. Integr. OMICS* 2 80–93. 10.5584/jiomics.v2i2.109

[B47] Palenik B. Brahamsha B. Larimer F. W. Land M. Hauser L. Chain P. (2003). The genome of a motile marine *Synechococcus*. *Nature* 424 1037–1042. 10.1038/nature01943 12917641

[B48] Perelman A. Uzan A. Hacohen D. Schwarz R. (2003). Oxidative stress in *Synechococcus* sp. strain PCC 7942: various mechanisms for H2O2 detoxification with different physiological roles. *J. Bacteriol.* 185 3654–3660. 10.1128/JB.185.12.3654-3660.2003 12775703 PMC156222

[B49] Pittera J. Humily F. Thorel M. Grulois D. Garczarek L. Six C. (2014). Connecting the thermal physiology and latitudinal niche partitioning in marine *Synechococcus*. *ISME J.* 8 1221–1236. 10.1038/ismej.2013.228 24401861 PMC4030225

[B50] Powers T. Noller H. F. (1995). A temperature-dependent conformational rearrangement in the ribosomal protein S4.16S rRNA complex. *J. Biol. Chem.* 270 1238–1242. 10.1074/jbc.270.3.1238 7836385

[B51] Qian X. Kumaraswamy G. K. Zhang S. Gates C. Ananyev G. M. Bryant D. A. (2016). Inactivation of nitrate reductase alters metabolic branching of carbohydrate fermentation in the cyanobacterium *Synechococcus* sp. Strain PCC 7002. *Biotechnol. Bioeng.* 113 979–988. 10.1002/bit.25862 26479976

[B52] Rabalais N. N. Turner R. E. Díaz R. J. Justić D. (2009). Global change and eutrophication of coastal waters. *ICES J. Mar. Sci.* 66 1528–1537. 10.1093/icesjms/fsp047

[B53] Rees A. Woodward M. Joint I. (1999). Measurement of nitrate and ammonium uptake at ambient concentrations in oligotrophic waters of the North-East Atlantic Ocean. *Mar. Ecol. Prog. Ser.* 187 295–300. 10.3354/meps187295

[B54] Ren Y. He Y. B. Lin Z. L. Zi J. Yang H. M. Zhang S. Y. (2018). Reagents for isobaric labeling peptides in quantitative proteomics. *Anal. Chem.* 90 12366–12371. 10.1021/acs.analchem.8b00321 30260629

[B55] Roberts E. W. Cai F. Kerfeld C. A. Cannon G. C. Heinhorst S. (2012). Isolation and characterization of the Prochlorococcus carboxysome reveal the presence of the novel shell protein CsoS1D. *J. Bacteriol.* 194 787–795. 10.1128/JB.06444-11 22155772 PMC3272956

[B56] Saito M. A. McIlvin M. R. Moran D. M. Goepfert T. J. DiTullio G. R. Post A. F. (2014). Multiple nutrient stresses at intersecting Pacific Ocean biomes detected by protein biomarkers. *Science* 345 1173–1177. 10.1126/science.1256450 25190794

[B57] Sajilata M. G. Singhal R. S. Kamat M. Y. (2008). The carotenoid pigment zeaxanthin—a review. *Compr. Rev. Food. Sci. Food. Saf.* 7 29–49. 10.1111/j.1541-4337.2007.00028.x

[B58] Sakamoto T. Delgaizo V. B. Bryant D. A. (1998). Growth on urea can trigger death and peroxidation of the Cyanobacterium *Synechococcus* sp. Strain PCC 7002. *Appl. Environ. Microbiol.* 64 2361–2366. 9647800 10.1128/aem.64.7.2361-2366.1998 PMC106396

[B59] Saunders G. F. Campbell L. L. (1966). Ribonucleic acid and ribosomes of *Bacillus stearothermophilus*. *J. Bacteriol.* 9 332–339. 5903099 10.1128/jb.91.1.332-339.1966 PMC315952

[B60] Savitski M. M. Wilhelm M. Hahne H. Kuster B. Bantscheff M. A. (2015). Scalable approach for protein false discovery rate estimation in large Proteomic data sets. *Mol. Cell. Proteomics.* 14 2394–2404. 10.1074/mcp.M114.046995 25987413 PMC4563723

[B61] Schäfer L. Sandmann M. Woitsch S. Sandmann G. (2006). Coordinate up-regulation of carotenoid biosynthesis as a response to light stress in *Synechococcus* PCC7942. *Plant Cell Environ.* 29 1349–1356. 10.1111/j.1365-3040.2006.01515.x 17080956

[B62] Schoeman D. S. Schlacher T. A. Jones A. R. Murray A. Huijbers C. M. Olds A. D. (2015). Edgingalong a warming coast: arange extension for acommon sandy beach crab. *PLoS One* 10:e0141976. 10.1371/journal.pone.0141976 26524471 PMC4629900

[B63] Six C. Thomas J. C. Brahamsha B. Lemoine Y. Partensky F. (2004). Photophysiology of the marine cyanobacterium *Synechococcus* sp. WH8102, a new model organism. *Aquat. Microb. Ecol.* 35 17–29. 10.3354/ame035017

[B64] Solomon C. M. Collier J. L. Berg G. M. Glibert P. M. (2010). Role of urea in microbial metabolism in aquatic systems: a biochemical and molecular review. *Aquat. Microb. Ecol.* 59 67–88. 10.3354/ame01390

[B65] Su Z. Mao F. Dam P. Wu H. Olman V. Paulsen I. T. (2006). Computational inference and experimental validation of the nitrogen assimilation regulatory network in cyanobacterium *Synechococcus* sp. WH 8102. *Nucleic Acids Res.* 34 1050–1065. 10.1093/nar/gkj496 16473855 PMC1363776

[B66] Ting C. S. Dusenbury K. H. Pryzant R. A. Higgins K. W. Pang C. J. Black C. E. (2015). The *Prochlorococcus* carbon dioxide-concentrating mechanism: evidence of carboxysome-associated heterogeneity. *Photosynth Res.* 123 45–60. 10.1007/s11120-014-0038-0 25193505

[B67] Tolonen A. C. Aach J. Lindell D. Johnson Z. I. Rector T. Steen R. (2006). Global gene expression of *Prochlorococcus* ecotypes in response to changes in nitrogen availability. *Mol. Syst. Biol.* 2 53. 10.1038/msb4100087 17016519 PMC1682016

[B68] Tukey J. W. (1977). *Exploratory data analysis*, Vol. 231 Reading, MA: Addison-Wesley.

[B69] Van Bogelen R. A. Neidhardt F. C. (1990). Ribosomes as sensors of heat and cold shock in *Escherichia coli*. *Proc. Natl Acad. Sci. U.S.A.* 87 5589–5593. 10.1073/pnas.87.15.5589 2198567 PMC54372

[B70] Varkey D. Mazard S. Ostrowski M. Tetu S. G. Haynes P. Paulsen I. T. (2016). Effects of low temperature on tropical and temperate isolates of marine *Synechococcus*. *ISME J.* 10 1252–1263. 10.1038/ismej.2015 26495993 PMC5029218

[B71] Warr S. R. C. Reed R. H. Stewart W. D. P. (1985). Carbohydrate accumulation in osmotically stressed cyanobacteria (blue-green algae): interactions of temperature and salinity. *New Phytol.* 100 285–295.

[B72] Wawrik B. Callaghan A. V. Bronk D. A. (2009). Use of inorganic and organic nitrogen by *Synechococcus* spp. and diatoms on the west Florida shelf as measured using stable isotope probing. *Appl. Environ. Microbiol.* 75 6662–6670. 10.1128/AEM.01002-09 19734334 PMC2772426

[B73] Wen B. Zhou R. Feng Q. Wang Q. Wang J. Liu S. (2014). IQuant: An automated pipeline for quantitative proteomics based upon isobaric tags. *Proteomics* 14 2280–2285. 10.1002/pmic.201300361 25069810

[B74] Woodson S. A. (2008). RNA folding and ribosome assembly. *Curr. Opin. Chem. Biol.* 12 667–673. 10.1016/j.cbpa.2008.09.024 18935976 PMC2651837

[B75] Wylie J. L. Worobec E. A. (1995). The OprB porin plays a central role in carbohydrate uptake in *Psedomonas aeruginosa*. *J. Bacteriol.* 177 3021–3026. 10.1128/jb.177.11.3021-3026.1995 7768797 PMC176988

[B76] Xing L. Sun L. Liu S. Li X. Zhang L. Yang H. (2017). IBT-based quantitative proteomics identifies potential regulatory proteins involved in pigmentation of purple sea cucumber, Apostichopus japonicus. *Comp. Biochem. Physiol. Part D Genomics Proteomics* 23 17–26. 10.1016/j.cbd.2017.05.004 28601631

[B77] Zeng Y. Huang X. G. Huang B. Q. Mi T. Z. (2016). Relationship between bacteria and phytoplankton during the giant jellyfish *Nemopilema nomurai* bloom in an oligotrophic temperature marine ecosystem. *Acta Oceanologica. Sinica.* 35 107–113. 10.1007/s13131-016-0894-x

[B78] Zhu Y. Graham J. E. Ludwig M. Xiong W. Alvey R. M. Shen G. (2010). Roles of xanthophyll carotenoids in protection against photoinhibition and oxidative stress in the cyanobacterium *Synechococcus* sp. strain PCC 7002. *Arch. Biochem. Biophys.* 504 86–99. 10.1016/j.abb.2010.07.007 20638360

[B79] Zwirglmaier K. Jardillier L. Ostrowski M. Mazard S. Garczarek L. Vaulot D. (2008). Global phylogeography of marine *Synechococcus* and Prochlorococcus reveals a distinct partitioning of lineages among oceanic biomes. *Environ. Microbiol.* 10 147–161. 10.1111/j.1462-2920.2007.01440.x 17900271

