# Supplementary material for: Proteomic Response to Rising Temperature in the Marine Cyanobacterium Synechococcus Grown in Different Nitrogen Sources
Source: Front Microbiol. 2019 Aug 23;10:1976. doi: 10.3389/fmicb.2019.01976 (PMC6716455; doi:10.3389/fmicb.2019.01976)
Supplement: Supplementary file 4 [file Data_Sheet_1.PDF]

SUPPORTING INFORMATION

Proteomic response to rising temperature in the marine cyanobacterium Synechococcus grown in different nitrogen sources

Yuan-Yuan Li[1], Xiao-Huang Chen[1], Cheng Xue[1], Hao Zhang[1], Geng Sun[1], Zhang-Xian Xie[1], Lin Lin[1], Da-Zhi Wang[1, 2]*

[1]State Key Laboratory of Marine Environmental Science/College of the Environment and Ecology, Xiamen University, Xiamen, 361005, China
[2]Key Laboratory of Marine Ecology & Environmental Sciences, China Academy of Sciences, Qingdao, 266071, China

*** Corresponding Authors**
Tel: 86-592-2186016. Email: dzwang@xmu.edu.cn.

Table of contents

Figure S1. Workflow for proteomic experiments using IBT labeling in this study. The numbers of “22”, “25” and “28” refer to temperature, and the symbols of “N” and “U” refer to nitrate and urea, respectively.

Figure S2. Coefficient of Variance (CV) distributions and ratio distributions between two biological replicates.

Figure S3. Distribution and the Venn diagrams of DEPs in *Synechococcus* WH8102 under different N sources and the increasing temperature conditions.

Figure S4. Functional classification and distributions of DEPs response to different N sources and the increasing temperature conditions.

Figure S5. GO classifications of DEPs response to different N sources at 25 ℃ and 28 ℃.

Table S1. The growth data of three formal experiments

Table S2. The information of peptide and protein identifications.

Table S3. The information of relative quantifications in each comparison.

Table S4. qPCR primers used in this study

Table S4. qPCR primers used in this study

	F	R
nrtA	ACTCGTCGATCCTGGTGTTC	TCCTTAGGCGGGAACCATTC
nirA	TTTCGGCGTATGGATCGGAG	TTGTTTCGTTCGCCGTTGTC
urtA2	GGTGGGTTCGGACTACGTTT	TGTTGATGATCACGCCTCCC
ureA	TCTTGGCAGAACGTCGACTC	TGTGCTGCCTTCCTGCATAA
glnA	AAGAGCTAGCGAAGATCGCC	GAAATCGTCGCTGAAGACGC
glsF	AAACCGGTGTGGTGGATCTC	TTCACGGTCCAGTTGTCGAG
16S rRNA	CATCATGCCCCTTACATCCT	AACTGAGCCACGGTTTATGG

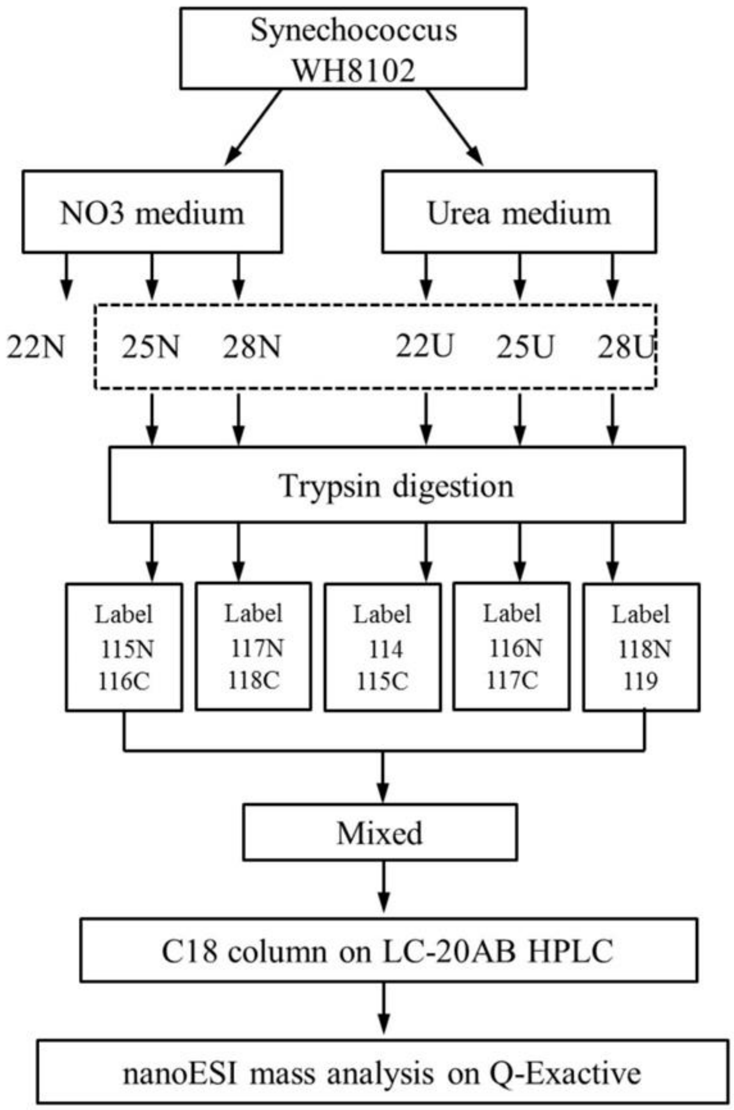

Figure S1. Workflow for proteomic experiments using IBT labeling in this study. The numbers of “22”, “25” and “28” refer to temperature, and the symbols of “N” and “U” refer to nitrate and urea, respectively.

22U

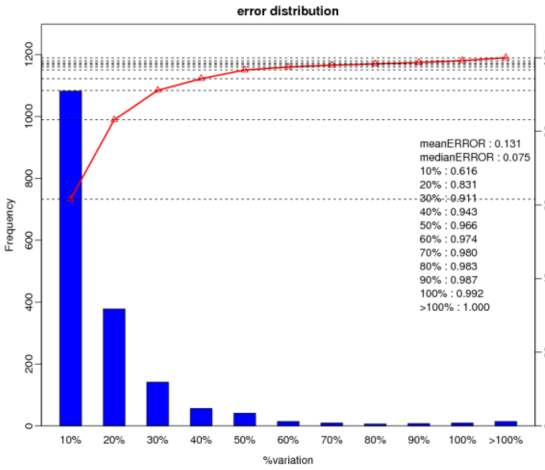
error distribution
Frequency
Cumulative%
%variation
10% 20% 30% 40% 50% 60% 70% 80% 90% 100% >100%
meanERROR : 0.131
medianERROR : 0.075
10% : 0.616
20% : 0.831
30% : 0.911
40% : 0.943
50% : 0.966
60% : 0.974
70% : 0.980
80% : 0.983
90% : 0.987
100% : 0.992
>100% : 1.000

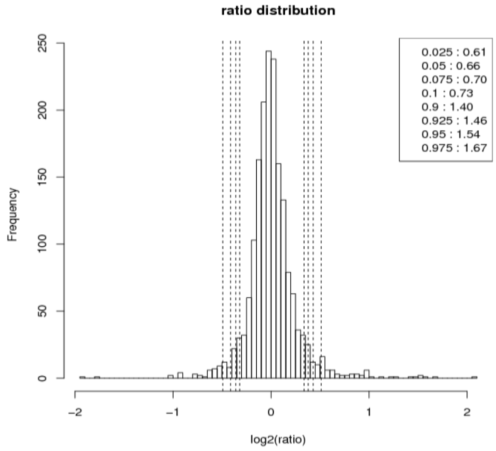
ratio distribution
Frequency
log2(ratio)
0.025 : 0.61
0.05 : 0.66
0.075 : 0.70
0.1 : 0.73
0.9 : 1.40
0.925 : 1.46
0.95 : 1.54
0.975 : 1.67

25N

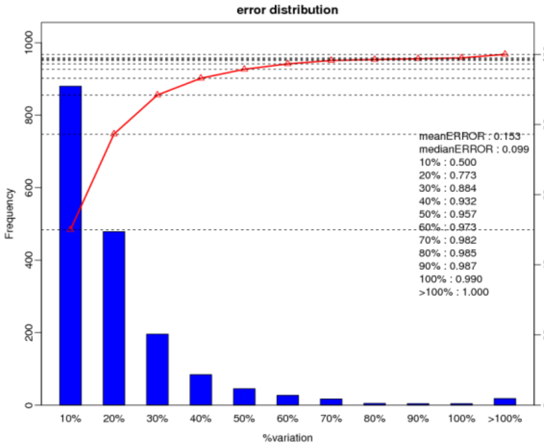
error distribution
Frequency
Cumulative%
%variation
10% 20% 30% 40% 50% 60% 70% 80% 90% 100% >100%
meanERROR : 0.153
medianERROR : 0.099
10% : 0.500
20% : 0.773
30% : 0.884
40% : 0.932
50% : 0.957
60% : 0.973
70% : 0.982
80% : 0.985
90% : 0.987
100% : 0.990
>100% : 1.000

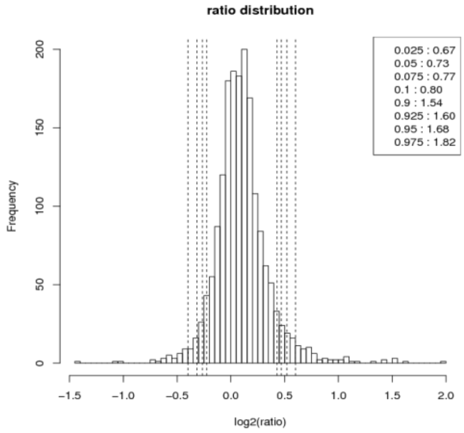
ratio distribution
Frequency
log2(ratio)
0.025 : 0.67
0.05 : 0.73
0.075 : 0.77
0.1 : 0.80
0.9 : 1.54
0.925 : 1.60
0.95 : 1.68
0.975 : 1.82

25U

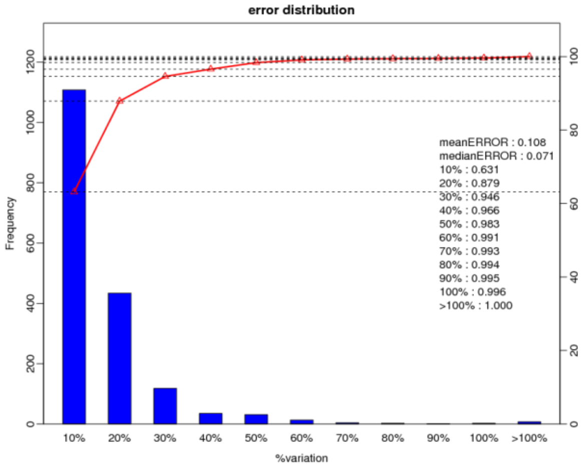
error distribution
Frequency
Cumulative%
%variation
10% 20% 30% 40% 50% 60% 70% 80% 90% 100% >100%
meanERROR : 0.108
medianERROR : 0.071
10% : 0.631
20% : 0.879
30% : 0.946
40% : 0.966
50% : 0.983
60% : 0.991
70% : 0.993
80% : 0.994
90% : 0.995
100% : 0.996
>100% : 1.000

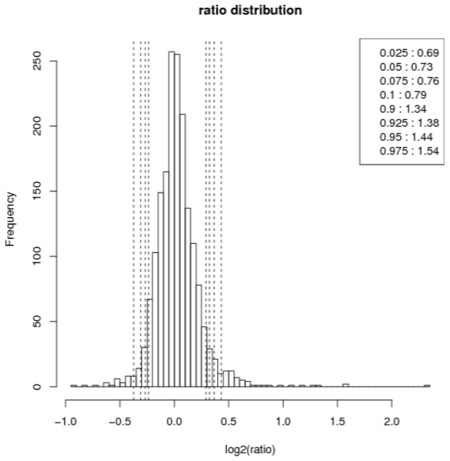
ratio distribution
Frequency
log2(ratio)
0.025 : 0.69
0.05 : 0.73
0.075 : 0.76
0.1 : 0.79
0.9 : 1.34
0.925 : 1.38
0.95 : 1.44
0.975 : 1.54

28N

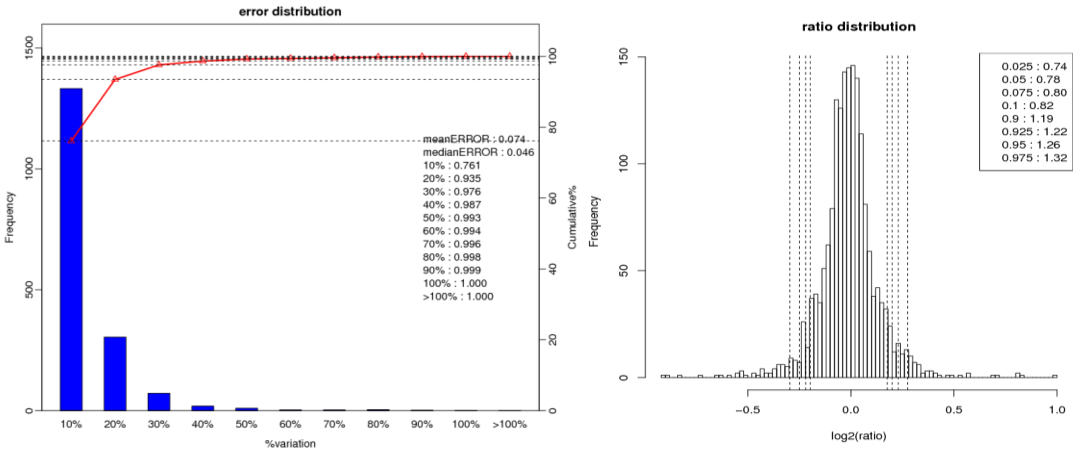

28U

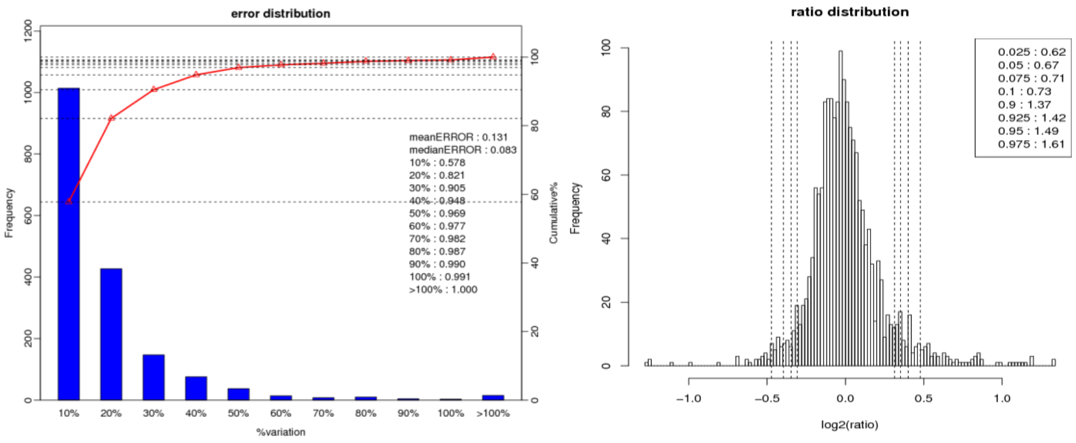

Figure S2. Coefficient of Variance (CV) distributions (Left) and ratio distributions (Right) between two biological replicates.

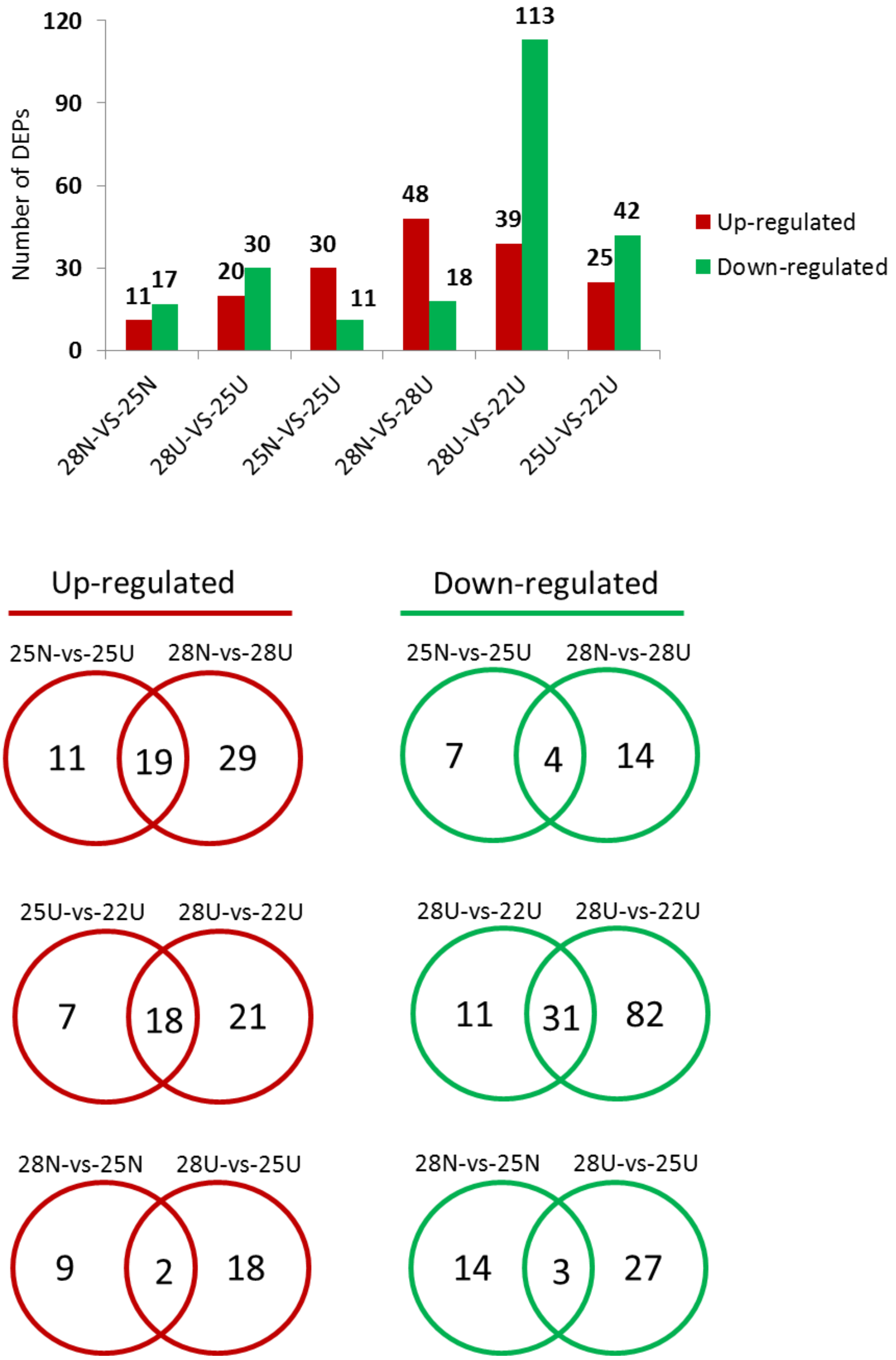

Figure S3. Distribution and the Venn diagrams of DEPs in *Synechococcus* WH8102 under different N sources and the increasing temperature conditions.

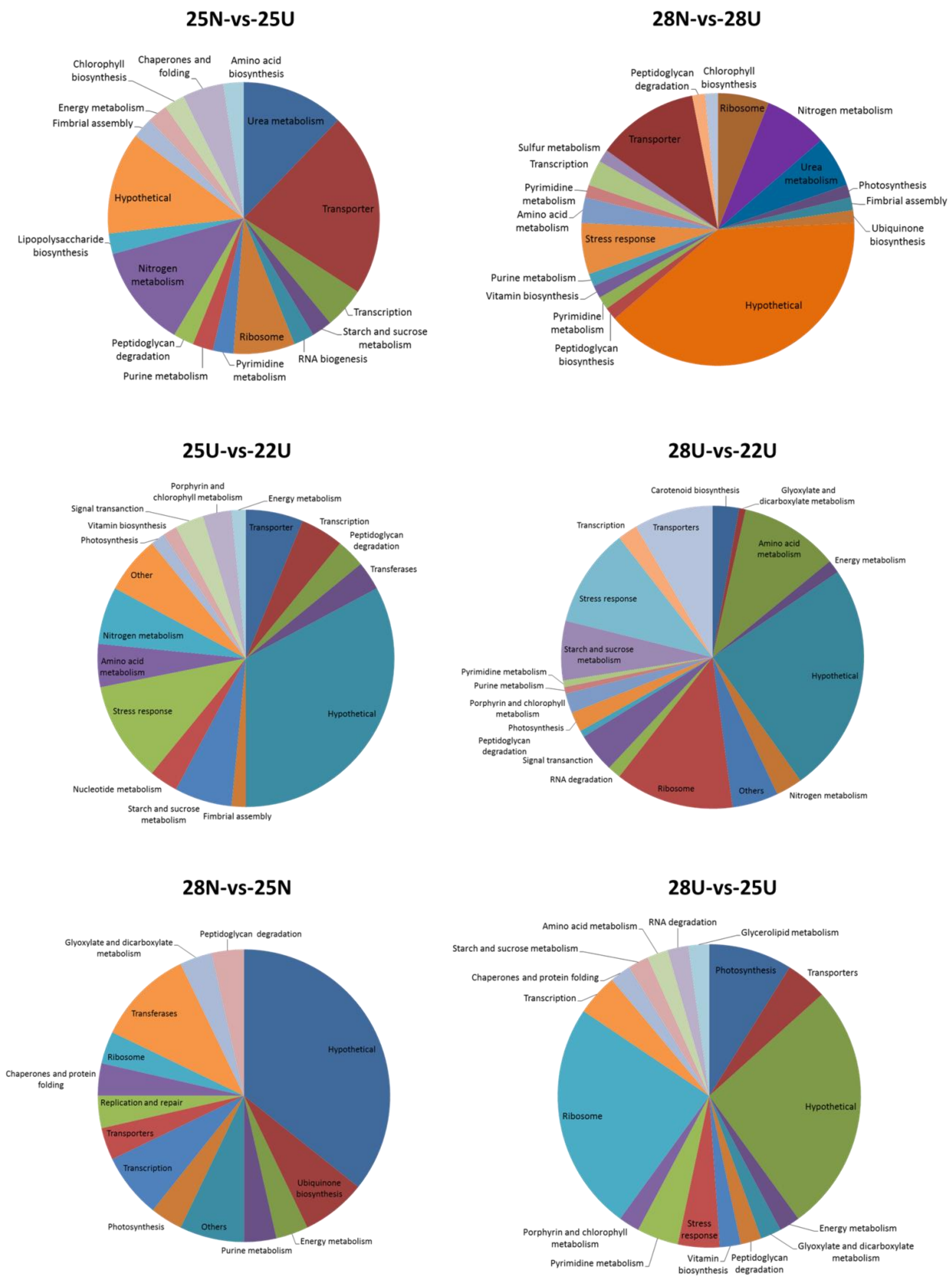

Figure S4. Functional classification and distributions of DEPs response to different N sources and the increasing temperature conditions.

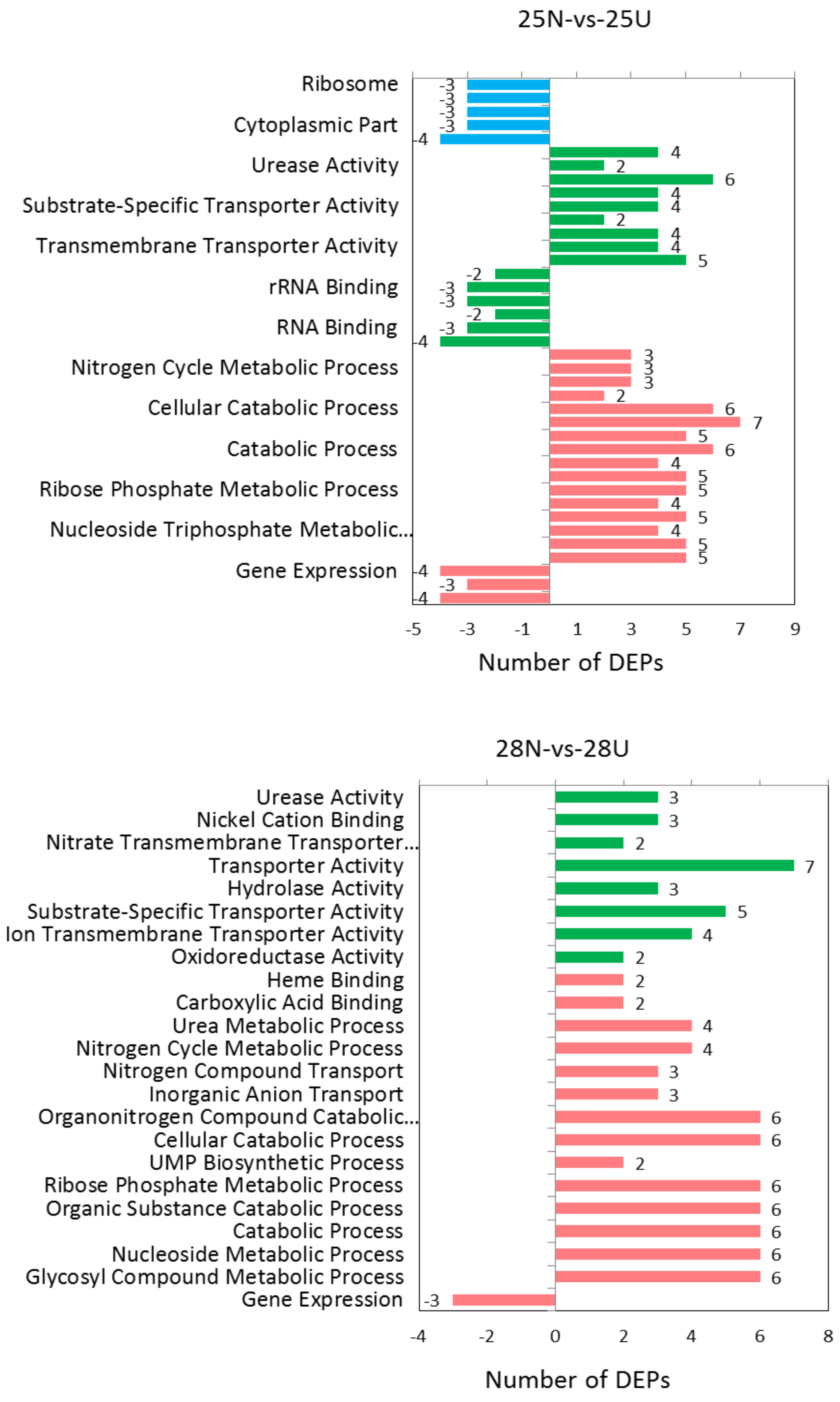

Figure S5. GO classifications of DEPs response to different N sources at 25 ℃ and 28 ℃.
